# Perceived communication skills and barriers among family medicine trainees and effectiveness of a communication workshop in Malaysia: A cross-sectional observational study with pre-post comparisons

**DOI:** 10.51866/oa.827

**Published:** 2026-08-25

**Authors:** Isriyanti Mohd Rafae, Irma Izani Mohammad Isa, Khubayb Ahmad Zamil, Mohammad Ariff Mohd Adip, Ni Jing Goi, Shi Ying Tan, Nurul Izzah Alipuddin, Ruziah Ibrahim, David Barron, Anthony G. Cummins, Sasikala Devi Amirthalingam

**Affiliations:** 1 Perdana University, Suite 9.3, Jalan Changkat Semantan, Damansara Height, Kuala Lumpur, Malaysia.; 2 Faculty of Medicine, University of Cyberjaya, Persiaran Bestari, Cyber 11, Cyberjaya, Selangor, Malaysia.; 3 College of Arts and Sciences, VinUniversity, Hanoi, Vietnam.; 4 Department of General Practice, University of Cork, Cork Ireland.; 5 IMU University, Clinical Campus, Jalan Rasah, Bukit Rasah, Seremban, Malaysia.

**Keywords:** Communication skills, Physicians-patient relationship

## Abstract

**Introduction:**

Effective communication skills are an integral part of healthcare provision, as they involve patient safety issues and shared decision-making. This study aimed to assess the perception of family medicine trainees (FMTs) about their communication skills and evaluate the effectiveness of a 2-day communication workshop in improving these skills.

**Methods:**

This cross-sectional observational study examined FMTs undergoing postgraduate training. A self-administered, validated questionnaire was utilised to assess conflict when dealing with patients, components of effective communication and barriers to good communication. The questionnaire was administered before and 6 months after the communication workshop.

**Results:**

Of 384 participants, most were aged 25–34 years (73.7%), women (75.8%), Malay (52.1%) and employed by the Ministry of Health Malaysia (82.0%). Approximately 82.8% opined that ≥75% of conflicts can be avoided through good communication skills. Among the 81 participants who completed the questionnaire before and after the workshop, the mean scores significantly increased post-workshop (P<0.05) for the following components of effective communication: communication of information and education, communication in complex situations, delivery of bad news and communication as part of team dynamics. The major barrier to practising effective communication was a lack of time (82.1%), while only a quarter of the participants (26.9%) indicated a lack of insight as a barrier.

**Conclusion:**

Communication workshops can help improve different aspects of communication among FMTs in Malaysia.

## Introduction

Communication skills play an indispensable role in daily living, especially among health professionals. Communication is a two-way process of conveying information, ideas, feelings or opinions between two or more persons, either verbally or non-verbally, using a medium.^1^ Verbal communication involves spoken words to convey thoughts, ideas and emotions.^2^ Non-verbal communication is a conscious or unconscious bodily response during communication, while paraverbal communication is the intonation of spoken words. Effective communication occurs when the message communicated by the sender is accurately translated or understood by the receiver. It is hence receiver-centric.

In primary care, physicians who deliver comprehensive healthcare services to individuals across all age groups within a family unit are commonly referred to as family doctors. Those who pursue and complete formal postgraduate training in family medicine are designated as family physicians; in the Malaysian context, they are specifically recognised as family medicine specialists. In this article, primary care doctors currently engaged in postgraduate training in family medicine are described as family medicine trainees (FMTs), and those who completed such training are referred to as family physicians.

Communication skills are part of the armamentarium for family physicians and other health professionals. In doctor–patient consultations, these skills are essential elements that ensure that both patients’ and doctors’ agendas are met.^3^ In primary care, effective communication skills are critical because general practitioners must deal with numerous uncertainties. Ineffective communication can lead to conflict, diagnostic failure and, occasionally, verbal and physical abuse.^4^ Family physicians deal with early presentations of patients’ illness and need to gather information on psycho-social background crucial in arriving at a diagnosis and facilitating successful patient management. Previous research has supported the importance of health professionals’ effective communication skills in improving both doctors’ and patients’ satisfaction with consultations, reducing litigation, increasing patients’ medication adherence and promoting patient safety.^5-8^ In modern undergraduate medical education, communication skills are embedded both horizontally and vertically within the curriculum. This approach equips graduates with high-quality communication skills, which involve not only taking history but also explaining diagnoses and treatments as well as communicating with other healthcare workers.^9,10^ Effective communication is a cornerstone of high-quality primary care, influencing patient satisfaction, adherence to treatment and clinical outcomes.^11^ Despite its recognised importance, communication training is often underemphasised in postgraduate medical education, particularly in resource-constrained or culturally diverse settings.^12-14^ Effective communication is one of the core domains that family physicians should possess. Embedding communication training into postgraduate family medicine curricula can help prevent future workplace conflicts by mitigating opposing values and perspectives through understanding, clearer treatment options and mutual agreement on certain decisions.^5,15^ There is much evidence in the literature that shows that communication gaps can cause conflict not only between patients and doctors but also within healthcare teams, leading to poorer patient outcomes.^16-18^

This study aimed to evaluate the effectiveness of a structured communication workshop, utilising an experiential learning approach designed for FMTs in Malaysia.^19^ The postgraduate training and workshop were undertaken by the Academy of Family Physicians of Malaysia (AFPM). The workshop aimed to enhance participants’ competencies in patient-centred communication, active listening, culturally sensitive interactions and shared decision-making. To assess its impact, we conducted a pre- and post-intervention survey, capturing self-reported changes in communication skills and conflicts in clinical practice. By examining the outcomes of this intervention, the study will contribute to ongoing efforts to strengthen clinical training and improve care quality in primary care settings.

## Methods

### Study design

This study employed an observational survey using a pre- and post-intervention design to evaluate the effectiveness of a communication workshop for postgraduate trainees in family medicine. Participants’ self-perceived communication competencies were assessed using a validated selfassessment tool adopted from the study by Baitha et al. at two time points: immediately before and 6 months after the workshop.^20^

### Study participants

The target participants included year-1 postgraduate trainees scheduled to undergo a communication workshop in family medicine. They were invited to participate in the study before the workshop; their participation was explicitly informed as voluntary and would not have any effects on their results or academic performance during or after the workshop. Participants in active clinical practice, currently undergoing postgraduate training and the workshop and willing to complete both pre- and post-workshop assessments were included. Ethical approval was secured from the Perdana University Institutional Review Board (PU-IRB) (ID: PU IRBHR0284). The president of the AFPM for the year 2019-2020 also granted permission for this research.

### Intervention

The communication workshop was designed to enhance core competencies in patient-centred communication, including active listening, empathy, shared decision-making and management of difficult conversations. It incorporated interactive lectures, role-play exercises and reflective discussions delivered over 2 full days by experienced speakers and facilitators. The workshop used the Calgary-Cambridge Guide to structure the communication training session.^1^ It was part of participants’ postgraduate curriculum. The workshop adopted interactive lectures and simulation of common communication in primary care through role plays, conducted over 2 days. Twelve role plays were used to allow participants to undergo experiential learning, although the workshop was conducted synchronously online due to the COVID-19 pandemic.^19,21,22^

### Assessment tool

Participants’ perceptions of their communication skills were measured using the self-administered validated questionnaire developed by Baitha et al. in 2019.^20^ The questionnaire consists of39 questions, which are divided into three sections (A-C) and use a 5-point Likert scale. The tool was initially developed for residents undergoing hospital training, requiring minor modifications and removal of two questions that were not suitable for the outpatient setting, with permission from the authors.

Section A comprises four questions that assess conflicts with patients and how communication manages or avoids such conflicts. Section B comprises 27 questions subdivided into five domains: components of effective communication (five items), communication of information and education (seven items), communication in complex situations (four items), delivery of bad news (five items) and communication as part of team dynamics (six items). Section C consists of eight questions that assess barriers to effective communication with patients.

### Data collection

Data were collected in August 2020 for the first cohort, July 2021 for the second cohort and January 2022 for the third cohort. Baseline data were gathered immediately before the workshop, labelled as the first phase. Follow-up data were obtained 6 months after the workshop, noted as the second phase. The questionnaire was sent electronically through Qualtrics. Unique identifiers were used to match pre- and post-workshop responses while maintaining confidentiality.

### Data analysis

Collected data were analysed using JASP version 16. Descriptive statistics were used to summarise participants’ socio-demographic and baseline characteristics. A paired sample t-test was conducted to compare pre- and post-intervention scores. Statistical significance was set at P<0.05.

## Results

The study compiled three cohorts of first-year postgraduate trainees who were scheduled for the communication workshop. The survey was performed for each cohort intake every 6 months for the three consecutive cohorts.

Among the first cohort, 130 participated in the workshop, and only 112 completed the first phase, yielding a response rate of 86%. The second cohort included 225 participants, among whom 167 completed the first phase, which yielded a response rate of 74%. Conversely, the third cohort comprised 170 participants, of whom 105 completed the first phase, yielding a response rate of 62%. The total number of first-phase responses was 384, which generated an average response rate of 73%; these responses were included in the descriptive analysis.

The number of second-phase responses after 6 months was smaller, with only 59, 43 and 23 responders from the first, second and third cohorts, respectively. Of the 125 responses, 81 second-phase responses were matched for analysis.

### Socio-demographic characteristics of the first-phase responders

The distribution of the socio-demographic characteristics of the participants is presented in Table 1. The participants were mostly aged 25–34 years (73.7%), with a mean age of 33.1 years. Approximately two-thirds were women (75.8%). The majority of the participants (82.0%) were family physicians working in the Ministry of Health, and more than half of them (52.1%) were Malay.

**Table 1:** Distribution of the socio-demographic characteristics.

Characteristic	Frequency (N=384)	Percentage
Age, year		
25-34	283	73.7
35-44	96	25.0
45-54	4	1.0
≥55	1	0.3
Sex		
Female	291	75.8
Male	91	23.7
Not disclosed	2	0.5
Race		
Malay	200	52.1
Indian	92	24.0
Chinese	73	19.0
Others	19	4.9
Current practice		
Ministry of Health	315	82.0
Private practice	62	16.1
Ministry of Higher Education	4	1.0
Ministry of Defence	3	0.8

### Section A: Conflicts with patients

Descriptive analysis was conducted on the first-phase responses. Section A of the questionnaire explored the participants’ experiences with workplace conflict. The findings indicated that 32.3% of the participants encountered minor conflicts either daily or at least once a week. Additionally, 9.7% reported major conflict with similar frequencies. For the purposes of this study, minor conflict was defined as an episode involving unwarranted arguments or debates escalating into heated discussions or shouting, without involving verbal abuse or physical aggression. In contrast, major verbal conflict was defined as the exchange of verbal abuse with patients or their attendants during the execution of clinical duties.

Most participants (66.4%) never exhibited any physical violence at the workplace, and 82.8% agreed that at least 75% of doctor-patient conflicts can be prevented through good communication practices. The results for section A are shown in Table 2.

**Table 2:** Frequency of experiencing conflicts with patients or attendants at the workplace.

	Frequency (percentage), N=384				
		Nearly daily	About once a week	About once a month	About once every 6 months	About once a year or less
Minor conflicts	29 (7.6)	95 (24.7)	89 (23.2)	80 (20.8)	91 (23.7)
Major conflicts	8 (2.1)	29 (7.6)	58 (15.1)	81 (21.1)	208 (54.2)
	**Four times or more**	**Three times**	**Two times**	**Once**	**None**
Physical violence so far	14 (3.6)	4 (1.0)	25 (6.5)	86 (22.4)	255 (66.4)
	**Almost all**	**About 75%**	**About 50%**	**About 25%**	**None**
Proportion of conflicts that can be avoided with good communication practices	170 (44.3)	148 (38.5)	56 (14.6)	5 (1.3)	5 (1.3)

### Section B: Effective communication skills and communication in different situations

Section B assessed the different components of communication, which are summarised in Table 3. Most participants (91.9%) reported often greeting their patients with a smile, doing namaste or saying hello and maintaining eye contact during conversations or interviews. However, only 41.1% always addressed their patients by name during history-taking, examination or interview. When providing information to or educating patients, more than 95% of the participants often used simple words and avoided medical jargon. Approximately 70%-80% explained the nature and progression of diseases, the necessity of expensive investigations and the effects and outcomes of treatment options and involved patients in decision-making.

**Table 3:** Effective communication skills and communication in different situations.

Item		Frequency (percentage), N=384				
		Always	Often	Sometimes	Occasionally	Rarely
Components of effective communication						
B1	While meeting a patient, I greet him/her warmly with a smile/do namaste/say hello.	259 (67.4)	94 (24.5)	23 (6.0)	5 (1.3)	3 (0.8)
B2	I prefer to address the patient by name during history-taking/examination/interview.	158 (41.1)	143 (37.2)	57 (14.8)	19 (4.9)	7 (1.8)
B3	I make eye contact during conversations or inter­views.	231 (60.2)	132 (34.4)	17 (4.4)	4 (1.0)	0 (0.0)
B4	While the patient is talking, I try to avoid any interruption, such as talking on the phone and checking messages.	216 (56.3)	128 (33.3)	36 (9.4)	4 (1.0)	0 (0.0)
B5	I pay attention to non-verbal cues like the gestures and facial expressions of the patient.	191 (49.7)	145 (37.8)	42 (10.9)	5 (1.3)	1 (0.3)
Communication of information and education						
B6	I ensure privacy while conducting interview/discussion sessions with the patient/attendant.	179 (46.6)	144 (37.5)	47 (12.2)	12 (3.1)	2 (0.5)
B7	I prefer simple language and avoid medical jargon and abbreviations.	231 (60.2)	135 (35.2)	17 (4.4)	1 (0.3)	0 (0.0)
B8	I explain the nature, course and prognosis (both short-term and long-term) of the disease in detail.	133 (34.6)	177 (46.1)	70 (18.2)	4 (1.0)	0 (0.0)
B9	I explain in detail the necessity and feasibility of expensive investigations and their effect on the course and outcome of the disease.	123 (32.0)	160 (41.7)	89 (23.2)	11 (2.9)	1 (0.3)
B10	I explain in detail various treatment options available and their effect on the course and outcome of the disease.	109 (28.4)	177 (46.1)	87 (22.7)	9 (2.3)	2 (0.5)
B11	I involve the patient in decision-making regarding the choice of investigation and treatment.	152 (39.6)	176 (45.8)	53 (13.8)	3 (0.8)	0 (0.0)
B12	Before concluding the interview, I ask the patient if he/she would like additional information.	126 (32.9)	141 (36.7)	94 (24.5)	18 (4.7)	5 (1.3)
Communication in complex situations						
B13	When the attendant gathers information from the internet or other sources, I try to answer their queries by giving better references.	87 (22.7)	170 (44.3)	109 (28.4)	13 (3.4)	5 (1.3)
B14	While discussing the patient’s progress, I emphasise the dynamic nature of the disease.	119 (31.0)	208 (54.2)	54 (14.1)	3 (0.8)	0 (0.0)
B15	I take consent from the patient/attendant myself.	207 (53.9)	133 (34.6)	35 (9.1	7 (1.8)	2 (0.5)
B16	I take consent from the patient/attendant after a detailed discussion.	170 (44.3)	146 (38.0)	51 (13.3)	13 (3.4)	4 (1.0)
Delivery of bad news						
B17	Before breaking bad news, I plan in advance and mentally rehearse the act of disclosure.	101 (26.3)	189 (49.2)	67 (17.4)	18 (4.7)	9 (2.3)
B18	Before breaking bad news, I tend to assess the relative’s/patient’s knowledge and attitude by asking open-ended questions.	120 (31.3)	159 (41.4)	79 (20.6)	22 (5.7)	4 (1.0)
B19	While breaking bad news, I tend to give information in small portions rather than doing it abruptly.	112 (29.2)	192 (50.0)	64 (16.7)	14 (3.6)	2 (0.5)
B20	After breaking bad news, I address/attend to the patient’s emotional reaction with full patience.	146 (38.0)	183 (47.7)	49 (12.8)	5 (1.3)	1 (0.3)
B21	After breaking bad news, I discuss the future plan of treatment with the patient and/or attendant.	174 (45.3)	171 (44.5)	35 (9.1)	4 (1.0)	0 (0.0)
Communication as part of team dynamics						
B22	While communicating with nurses, paramedical staff and other supporting staff, I display appropriate courtesy.	232 (60.4)	138 (35.9)	13 (3.4)	0 (0.0)	1 (0.3)
B23	While communicating with nurses, paramedical staff and other supporting staff, I highlight that their roles and responsibilities are equally important.	209 (54.4)	147 (38.3)	24 (6.3)	3 (0.8)	1 (0.3)
B24	I avoid criticising colleagues or having debates in front of patients or attendants.	249 (64.8)	117 (30.5)	15 (3.9)	2 (0.5)	1 (0.3)
B25	To motivate nurses, paramedical staff and other supporting staff, I teach them the principles of management of commonly encountered diseases in the ward.	130 (33.9)	168 (43.8)	77 (20.1)	8 (2.1)	1 (0.3)
B26	As part of giving feedback, I regularly express appreciation for nurses, paramedical staff and other supporting staff.	181 (47.1)	159 (41.4)	41 (10.7)	3 (0.8)	0 (0.0)
B27	As part of giving feedback, I do not hesitate to give positive criticism/constructive suggestions to my subordinates and supporting healthcare staff.	125 (32.6)	163 (42.4)	79 (20.6)	10 (2.6)	7 (1.8)

A substantial proportion of the participants reported routinely involving patients in clinical decision-making. Specifically, 85.7% indicated that they often or always engage patients during the decision-making process. Furthermore, 70% of the participants stated that they typically inquire whether patients require additional information prior to concluding consultations.

Approximately 88.5% of the participants indicated obtaining consent from patients or attendants by themselves, with 82.3% often doing so after a detailed discussion. When patients sought information from the internet, around 20% of the participants consistently provided more reliable references to guide their understanding. In preparation for delivering bad news, more than 70% of the participants often planned, mentally rehearsed and assessed relatives’ or patients’ knowledge, attitude and expectations. Following the delivery of bad news, more than 85% addressed patients’ emotional reaction with full patience and discussed their plan.

In terms of communication as part of team dynamics, 95% of the participants avoided criticising their colleagues in front of patients and displayed courtesy when communicating with nurses and other staff. Approximately 80% often taught nurses and other staff about disease management principles and provided them with constructive feedback.

### Section C: Barriers to good communication

The findings for section C, which examined barriers to effective communication, are summarised in Table 4. The most cited barrier was a lack of time, which 80.7% of the participants reported as a major cause of disruption to good communication with patients. A substantial proportion (77.4%) indicated that human factors, specifically stress and fatigue, would make it difficult to implement good communication practices. Additionally, 75.5% of the participants agreed that infrastructural deficiencies hinder communication efforts. Approximately 69.1% also agreed that barriers to communication include difficulty in understanding patients’ language, a lack of subject knowledge, long working hours and a lack of training in communication skills. Notably, only 27.6% of the participants identified a lack of insight as a barrier to effective communication.

**Table 4:** Barriers to good communication.

Item		Frequency (percentage), N=384				
		Strongly agree n (%)	Somewhat agree n (%)	Neither agree nor disagree n (%)	Somewhat disagree n (%)	Strongly disagree n (%)
C1	Lack of insight	53 (13.8)	53 (13.8)	42 (10.9)	70 (18.2)	166 (43.2)
C2	Lack of time	163 (42.4)	147 (38.3)	25 (6.5)	27 (7.0)	22 (5.7)
C3	Difficulty in understanding the patient’s language	97 (25.3)	168 (43.8)	57 (14.8)	43 (11.2)	19 (4.9)
C4	Human failings such as stress and fatigue	81 (21.1)	216 (56.3)	35 (9.1)	41 (10.7)	11 (2.9)
C5	Infrastructural deficits, such as a lack of a proper place for discussion and overcrowding	121 (31.5)	169 (44.0)	41 (10.7)	34 (8.9)	19 (4.9)
C6	Long working hours	93 (24.2)	151 (39.3)	75 (19.5)	49 (12.8)	16 (4.2)
C7	Lack of subject knowledge required for fully explaining the modalities of diagnosis, treatment options or prognosis	63 (16.4)	185 (48.2)	65 (16.9)	50 (13.0)	21 (5.5)
C8	Lack of training in communication skills	61 (15.9)	184 (47.9)	75 (19.5)	49 (12.8)	15 (3.9)

### Effectiveness of the communication workshop

The effectiveness of the communication workshop was assessed by analysing 81 matched pre- and post-intervention responses using the paired t-test, as presented in Table 5.

**Table 5:** Communication skills pre- and post-workshop.

Mean ± standard deviation			P-value
Item^*^	Pre-workshop	Post-workshop	
Components of effective communication (theme 1)	4.447±0.462	4.444±0.428	0.954
B5	4.247±0.845	4.531±0.614	0.003
Communication of information and education (theme 2)	4.206±0.532	4.344±0.512	0.006
B6	4.198±0.872	4.407±0.755	0.014
B8	4.198±0.765	4.383±0.624	0.024
Communication in complex situations (theme 3)	4.269±0.600	4.441±0.504	0.007
B16	4.370±0.782	4.654±0.528	0.003
Delivery of bad news (theme 4)	4.151±0.594	4.304±0.564	0.013
B18	4.000±0.949	4.222±0.775	0.043
B19	4.074±0.703	4.296±0.766	0.024
Communication as part of team dynamics (theme 5)	4.356±0.473	4.506±0.443	0.001
B22	4.556±0.592	4.778±0.418	0.001
B24	4.556±0.570	4.728±0.475	0.007
B25	4.074±0.818	4.309±0.718	0.014

* Item with significant improvements post-workshop only.

For theme 1, which encompassed the components of effective communication (B1-B5), the overall score did not reach statistical significance (B1-B5; 4.447±0.462 vs 4.444±0.428, P=0.954). However, a significant improvement was observed in attention to verbal cues (B5; 4.247±0.845 vs 4.531±0.614, P=0.003).

In theme 2, which focused on communicating information and educating patients and their relatives (B6-B12), the mean score for the overall items increased significantly from 4.206±0.532 to 4.344±0.512 (P=0.006). The post-workshop scores for ensuring patient privacy during consultations significantly increased (B6; 4.198±0.872 vs 4.407±0.755, P=0.014). A similar increment was observed for providing detailed explanations regarding the nature, progression and prognosis of illness (B8; 4.198±0.765 vs 4.383±0.624, P=0.024).

For theme 3 (communication in complex situations; B13-B16), the mean score increased significantly from 4.269±0.600 to 4.441±0.504, with a P-value of 0.007. The score for obtaining consent from patients or attendants after a detailed discussion also significantly increased (B16; 4.370±0.782 vs 4.654±0.528, P=0.003). In contrast, although the score for shared decision-making increased, it was not significant.

For theme 4, which involved delivering bad news, the score increased significantly after the workshop (B17–B21; 4.151±0.594 vs 4.304±0.564, P=0.003). Two other items yielded significantly increased scores: assessing relatives’ or patients’ knowledge and attitude by asking open-ended questions (B18; 4.000±0.949 vs 4.222±0.775, P=0.043) and providing information in small portions rather than doing it abruptly (B19; 4.074±0.703 vs 4.296±0.766, P=0.024).

Finally, the best overall improvement was noted in theme 5 (communication as part of team dynamics; B22–B27), with the mean score increasing from 4.356±0.473 to 4.506±0.443 (P=0.001). The items that yielded significantly increased scores included displaying appropriate courtesy when communicating with nurses and other staff (B22; 4.556±0.592 vs 4.778±0.418, P=0.001), avoiding criticism of colleagues or debates in front of patients (B24; 4.556±0.570 vs 4.728±0.475, P=0.007) and motivating nurses, paramedics and supporting staff (B25; 4.074±0.818 vs 4.309±0.718, P=0.014).

## Discussion

This study demonstrated a positive impact of the communication workshop on the communication competencies perceived by the FMTs. Although the FMTs already possessed good communication skills before the workshop (mean score of4.00–4.56/5.00), the post-workshop assessments revealed further enhancement in their communication skills. Prominently, improvements were observed in the domain of providing patient education and communicating information.

Educating patients includes explaining details of their illness, the ability to effectively convey the prognosis and nature of the illness and the importance of investigation and treatment options. This may increase the understanding of patients about their condition and promote shared decisionmaking for optimal patient care.^8,23,24^ The workshop also improved communication with other healthcare professionals in the workplace, including fellow doctors, nurses, paramedical staff and supporting staff. This aligns with the findings of D’Antonio et al. in their quality improvement project in a Pennsylvania hospital, in which standardising and improving the quality of verbal and non-verbal communication skills improved patient outcomes.^25^ Another study showed that communication training yielded an overall positive impact on teamwork culture, patient satisfaction and patient care.^26^

Shared decision-making was explicitly addressed as a core component of the workshop. Incorporating the need for patient-centric, rather than physicians’ agenda, during consultations is an important part of effective communication. However, the workshop did not improve the participants’ perceptions of shared decision-making in this study. This finding contrasts with descriptive data indicating that over 80% of FMTs routinely involve patients in clinical decision-making. Such discrepancy may reflect systemic challenges within the Malaysian healthcare landscape, in which there are many barriers to shared decision-making, including cultural and language diversity, time constraint, limited health literacy and inadequate access to health information, along with persistence of paternalistic attitudes among healthcare providers including physicians.^27^

Major conflicts, defined in this study as incidents involving verbal abuse, were reported by 54.2% of the FMTs to have occurred at least once within the past year. This finding aligns with data from the National Study of Australian General Practitioners, which indicated that 57% of respondents experienced verbal abuse over a similar 12-month period, with younger practitioners disproportionately affected by such encounters.^28^ In China, 72.4% of hospital-based physicians have reported exposure to workplace violence.^29^ The higher rate of such incidents in the hospital setting is likely attributable to the demanding nature of the clinical duties of FMTs who often work extended hours. Similarly, in India, major conflicts and physical violence faced by trainee or resident doctors have mostly occurred during out-of-office hours, where the expectation of treatment is heightened or when health professionals are tired or burnt out.^30^

Over half of the participants reported experiencing minor conflicts, defined as incidents leading to shouting and unwarranted arguments not leading to verbal abuse for at least once a week.

Previous research has identified several contributing factors to such conflicts, including long waiting times (17%–32.5%), communication barriers (23.5%), unmet service needs (36%–72%) and overcrowding (33%–66%).^31^ Most previous reports support our conclusion that effective communication is important to avoid these conflicts. This further highlights the importance of communication training among primary care doctors. Notably, prior studies have suggested that fostering collaboration and empathy during patient interactions can transform potentially adversarial encounters into constructive and therapeutic engagement.^32^

In this study, most FMTs agreed that difficulty in understanding patients’ language, a lack of time and physical barriers hinder effective communication. Because Malaysia is multi-racial and has a large foreign workforce, doctors need to be fluent in multiple languages.^33^ In the public healthcare system in Malaysia, large numbers of patients are seen every day, leading to long waiting times and often limited privacy due to the lack of individual confidential consultation rooms, which add to the challenges of implementing effective communication. The perceived barriers to effective communication can be addressed by making changes to the infrastructure and system and providing training for family physicians.

The parallel pathway to postgraduate training in family medicine appears to be a preferred option among female physicians. In Malaysia, over 70% of family medicine specialists are women.^34^, This trend may be partially attributed to the competing demands of professional responsibilities, marital commitments and childcare.^35^

A comparable pre- and post-intervention study conducted among first-year postgraduate students in India evaluated the impact of a brief communication training module delivered over 4 consecutive days (1.5 hours per day). The intervention yielded significant improvements in communication competency, with mean post-test scores of 25.19±3.76 based on self-assessment and 24.26±2.94 based on faculty evaluation (P<0.001).^6^ However, these findings do not substantiate the effectiveness of shorter interventions – such as a 2-day workshop – in fostering sustained behavioural change. Emerging evidence suggests that ongoing mentorship and structured supervision by experienced family physicians are more effective in cultivating professional growth and supporting the longterm development of communication competency among future family medicine specialists.^36,37^

A more objective evaluation of communication training or interventions may require assessing clinical outcomes, such as patient satisfaction and reductions in complaints or litigation, as these provide a more robust measure of training effectiveness. Currently, evaluation remains at level 1 of the Kirkpatrick model, focusing primarily on participants’ perceptions. A demonstrable improvement in perceived skills may elevate the assessment to level 2, suggesting that actual learning has taken place.^38^ In the future, communication skills may need to be evaluated both formatively and summatively by trained facilitators or clinical supervisors in a long-term supportive training environment, preferably in the workplace.^36,39^ To strengthen the evidence base, future research on patients’ perceptions aiming to extend evaluation to level 3 (behaviour) by examining the transfer of communication skills into clinical practice is needed. Ideally, evaluation to level 4 (results) by assessing the impact of improved communication on patient outcomes and healthcare delivery can be undertaken.

This study presents several limitations. First, it was conducted during the COVID-19 pandemic, necessitating the exclusive use of an online platform for data collection and workshop delivery. This constraint contributes to a high attrition rate, especially for the second phase. Second, the reliance on the self-assessment by the FMTs introduces social desirability bias, as the participants may have under- or overestimated their communication competencies. To mitigate this bias in future research, researchers should incorporate patients’ perspectives to achieve a more objective and balanced evaluation. Third, the questionnaire was adapted for the assessment of resident doctors and may therefore not fully represent the situation in the primary care setting. In the future, a qualitative study can be conducted to obtain a better understanding of the communication skills of FMTs.

A key strength of this study lies in its design, which enabled the evaluation of the perceived communication skills of postgraduate trainees both before and after the workshop. The inclusion of three distinct cohorts during the first phase provided a robust and nuanced overview of how the

FMTs assessed their own communication competencies, enhancing the depth and generalisability of the findings.

## Conclusion

In conclusion, the FMTs demonstrated a strong baseline in their communication competencies, which were further enhanced following participation in the 2-day communication workshop. Minor conflicts were frequently reported in outpatient settings, whereas major verbal confrontations and physical violence were comparatively rare. A lack of time remained a predominant barrier to effective communication, likely due to high patient volumes, typically present in outpatient settings. Future research should consider incorporating perspectives from key healthcare stakeholders, particularly patients, to gain a more comprehensive understanding of communication dynamics and their impact on healthcare delivery.^40^

## Data sharing statement

Our data are available only upon request and will not be shared publicly under the data sharing plan.

**How does this paper make a difference in general practice?**

- Communication skills are the first domain in general practice identified in the RACGP curriculum that general practitioners must master. This descriptive study provided data on the communication skills of family medicine trainees.
- We explored the perceived barriers to effective communication faced by family medicine trainees. Identifying these barriers will help address the underlying issues and improve patient–physician communication, thereby strengthening the therapeutic relationship.
- The survey was conducted before and 6 months after the communication workshop to gauge whether the intervention was effective.
